# Producing routine malaria data: an exploration of the micro-practices and processes shaping routine malaria data quality in frontline health facilities in Kenya

**DOI:** 10.1186/s12936-019-3061-y

**Published:** 2019-12-16

**Authors:** George Okello, Sassy Molyneux, Scholastica Zakayo, Rene Gerrets, Caroline Jones

**Affiliations:** 1 Kenya Medical Research Institute-Wellcome Trust Research Programme, P.O Box 230, 80108 Kilifi, Kenya; 2 Centre for Tropical Medicine and Global Health, University of Oxford, Oxford, UK; 3 Department of Anthropology, University of Amsterdam, Amsterdam, The Netherlands

**Keywords:** Health information system, Malaria surveillance, Routine data

## Abstract

**Background:**

Routine health information systems can provide near real-time data for malaria programme management, monitoring and evaluation, and surveillance. There are widespread concerns about the quality of the malaria data generated through routine information systems in many low-income countries. However, there has been little careful examination of micro-level practices of data collection which are central to the production of routine malaria data.

**Methods:**

Drawing on fieldwork conducted in two malaria endemic sub-counties in Kenya, this study examined the processes and practices that shape routine malaria data generation at frontline health facilities. The study employed ethnographic methods—including observations, records review, and interviews—over 18-months in four frontline health facilities and two sub-county health records offices. Data were analysed using a thematic analysis approach.

**Results:**

Malaria data generation was influenced by a range of factors including human resource shortages, tool design, and stock-out of data collection tools. Most of the challenges encountered by health workers in routine malaria data generation had their roots in wider system issues and at the national level where the framing of indicators and development of data collection tools takes place. In response to these challenges, health workers adopted various coping mechanisms such as informal task shifting and use of improvised tools. While these initiatives sustained the data collection process, they also had considerable implications for the data recorded and led to discrepancies in data that were recorded in primary registers. These discrepancies were concealed in aggregated monthly reports that were subsequently entered into the District Health Information Software 2.

**Conclusion:**

Challenges to routine malaria data generation at frontline health facilities are not malaria or health information systems specific; they reflect wider health system weaknesses. Any interventions seeking to improve routine malaria data generation must look beyond just malaria or health information system initiatives and include consideration of the broader contextual factors that shape malaria data generation.

## Background

Routine health information systems (HIS) are crucial for effective malaria control and elimination [1]. Where functional, these systems can provide near real time data on malaria cases reported rather than relying on mathematically modelled estimates of malaria burden [2]. Such data are important for tracking the progress of malaria control, advocating for adequate investments, supporting appropriate allocation and targeting of resources, and for disease surveillance [3]. In many countries and particularly in sub-Saharan Africa where malaria is endemic, routine HIS are often weak and there are widespread concerns about the quality and utility of the malaria data generated [4–7]. Despite recognized weaknesses in routine HIS, the renewed drive towards malaria elimination has reinvigorated the interest in malaria data generated through these systems. For instance, the Global Technical Strategy for Malaria 2016–2030 stresses the need for sufficient investment in the management and use of data from routine health information systems to support programme planning, implementation and evaluation [3].

In Kenya, mirroring the global interest in malaria surveillance, an objective of the National Malaria Strategy 2019–2023 is to strengthen malaria surveillance and use of information to improve decision making for programme performance [8]. To standardize routine health data generation in the country, the Ministry of Health has developed standard data collection registers and reporting forms which all public and private health facilities are required to use in data collection and reporting. Routine data collected at frontline health facilities are subsequently collated at sub-county level and reported through the District Health Information Software (DHIS2), a web-based health information system for the collation and reporting of routine health and management data launched in 2011 [9]. In line with the devolved structure of governance, county governments are now directly responsible for monitoring and evaluation of all health services in their counties, including the collection and collation of routine health information, and analysis and dissemination of these data. To improve the quality of routine health information, regular support supervision visits and data quality audits are recommended at the health facility and sub-county levels where data collection, collation and aggregation takes place. Data quality audits are conducted with technical support from the national government.

Despite attempts to improve the quality of routine malaria data, recent assessments of Kenya’s HIS have identified persistent data quality issues with routine malaria data that have implications on the validity of malaria indicators constructed using such data. Some of the documented data quality issues include: underreporting or overreporting of malaria cases and treatments; misclassification of malaria cases in data collection registers; and missing data or reporting forms [10–12]. Data quality audits (DQAs) have also highlighted various organizational (e.g. stock-out of tools and human resources shortages), social and behavioural (e.g. data recording practices) and technical factors (e.g. tools and indicators) that undermine health data collection in the country in general [13].

However, as is the case with most assessments of the routine HIS, these DQAs concentrate primarily on assessing the quantitative dimensions of data quality (i.e. completeness, timeliness, and accuracy) [14]. In addition, they are primarily cross sectional and focused on the data produced, revealing little about the underlying practices and processes that contribute to data quality issues, particularly at the frontline health facility where data are collected. Few studies have examined the micro-level practices of data collection that are central to the production of routine malaria data [5–7]. This study draws on empirical data collected as part of a broader study investigating how data for constructing routine malaria indicators are produced at the local level to examine the processes and practices that shape routine malaria data quality at frontline health facilities in Kenya. Understanding how malaria data are generated at this level, and the implications of these micro-level practices activities have on data quality, is crucial for the on-going development of systems that can improve the outcome of the data collection process.

**Fig. 1:**
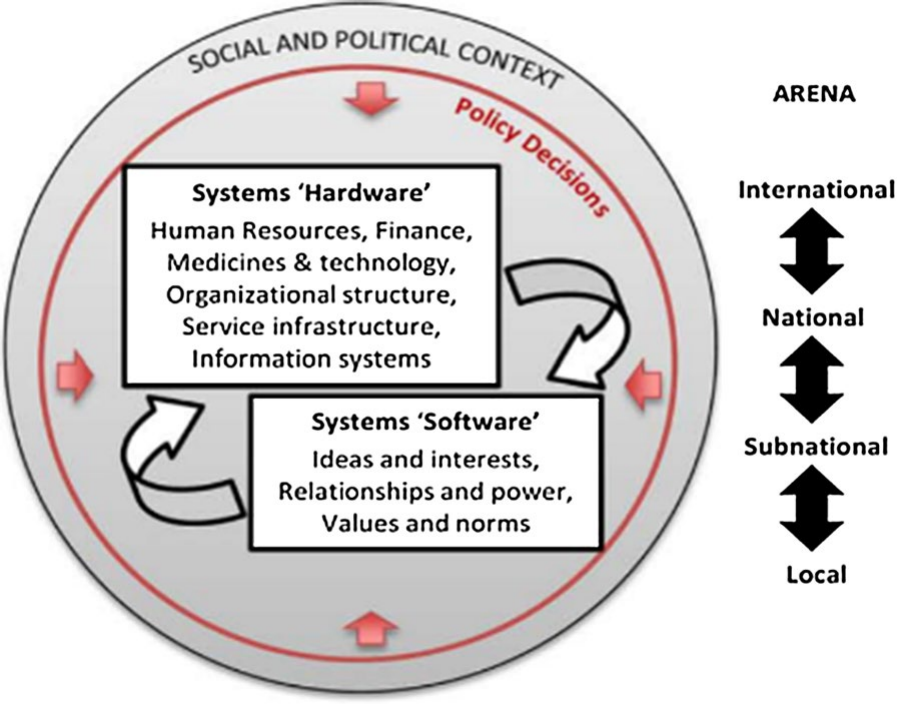
Sheikh et al’s framework for understanding the health system

To examine the micro-level practices and processes of data collection at frontline health facilities, this study draws on the framework by Sheikh et al. which considers the roles of the individual involved in activities of health provision, utilization and governance, and how systems respectively shape and are shaped by their actions and behaviour [15]. It explores how health workers involved in routine data generation draw on their interests, relationships, and power (systems ‘software’) to overcome various system ‘hardware’ constraints (Fig. 1) and in the process, keep the data pipeline flowing.

The study also draws on VeneKlasen and Miller’s conceptualization of power; examining how health workers mobilize different forms of power to address the challenges they face. These authors describe four forms of power: *power over; power to; power with;* and *power within* (Table 1) [16].

**Table 1:** VeneKlasen and Miller’s forms of power

Forms of power	Definition
Power over	Involves taking power from someone else, then using it to dominate or to prevent others from gaining it (normally has negative connotations)
Power within	Has to do with a person’s self-worth and self-knowledge (i.e. ability to recognize individual differences while respecting others)
Power to	Refers to the unique potential of every person to shape his or her life and world
Power with	Involves finding common ground among different actors and building collective strength

## Methods

### Setting

The study was conducted in two of the fourteen malaria endemic counties in Kenya where core malaria prevention, diagnosis and treatment interventions have been scaled up over past decade [8]. One county was located in the coast region (malaria prevalence 8% in 2015) and the other in the lake region (malaria prevalence 27% in 2015) [17]. In each county, one sub-county (equivalent to a district) was purposively selected based on their location to study sub-county health management offices. In each sub-county, two frontline health facilities were selected (a health centre and a dispensary) to examine the production of routine malaria data. Dispensaries and health centres have varying levels of staffing and workload. For instance, while health centres serve an average population of 30,000 people, dispensaries serve an average population of 10,000 people [18]. Sampling of health facilities within the sub-counties therefore aimed to capture variation based on facility size and workload. The selection of health facilities was also informed by their accessibility (i.e. those close to the sub-county health management offices vs those in remote locations), the availability of a working laboratory capable of conducting malaria microscopy and having no record of previous involvement in research activities.

### Data collection

Data collection was undertaken by two experienced qualitative researchers (GO and SZ) between January 2015 and August 2016. The study employed an ethnographic approach involving longitudinal observations, records review, and interviews. Observations (both participant and non- participant) at the health facility level focused on understanding malaria data generation and reporting practices in the laboratory; outpatient clinics; pharmacies; and antenatal care clinics. Malaria data collection registers and monthly reporting forms were retrospectively reviewed at the start of field work (for the past 3 months) to document malaria data recording and reporting practices, and to understand how malaria data travelled from service delivery areas into monthly reports and eventually into the DHIS2. Formal interviews were conducted with frontline staff (n = 13), sub-county managers (n = 9) and national level policy makers (n = 5) to gain their perspectives on malaria data generation processes and practices. All interviews and meetings were conducted in both English and Kiswahili and took place in locations that were convenient to participants. Where consent was provided for digital audio recording, interviews were audio-recorded and subsequently transcribed and translated. Following an initial analysis of data collected, preliminary feedback meetings were held with health workers in all four facilities, and with a larger group of health workers drawn from other facilities in the two sub-counties (n = 35) and their managers (n = 17). These feedback meetings were an opportunity to share and validate preliminary findings and gather new data and understanding.

### Data analysis

Interview transcripts and field notes were imported into NVivo 10 for data management and analysis. Data analysis was undertaken concurrently with data collection and was guided by the thematic content analysis approach [19]. This involved reading and familiarization with the data and development of an initial coding framework which was constantly reviewed as more data were collected and new categories emerged. The final coding framework developed at the end of data collection was used to code the entire dataset. The final step in the data analysis process involved looking for patterns and relationships between themes and sub-themes and relating these to Sheikh’s framework (Fig. 1) and with the wider literature.

## Results

The results are divided into three sections. The first provides a brief description of the four study facilities. The second explains how routine data on malaria diagnosis are generated at frontline health facilities, and highlights some of the data quality issues that were observed during records review, while the third section focuses on a description of the practices and processes that shape data collection and recording practices and identification of the underlying systems factor influences.

### Description of the four study facilities

The four health facilities (referred to in this paper as facility A, B, C, and D) provided similar curative, preventive and promotive services but differed in physical infrastructure, staffing and workload (Table 2). Generally, facility A was the largest and busiest. There were more outpatient confirmed malaria cases in facility B and C which were located in the lake region sub-county where malaria prevalence is highest [17]. Nursing officers were the main cadre of staff found in all four facilities (Table 2). There was a shortage of other recommended cadres of staff such as clinical officers, laboratory technologists and pharmaceutical technologists and health records officers in all four facilities. To fill the staffing gaps, health facility management committees used locally generated resources (such as user fees for laboratory services) and other discretionary funds received from the national government to hire laboratory technologists and other support staff (such as nurse aids, data clerks, drug dispensers and cashiers). Although support staff’s roles were mainly auxiliary, there were instances when these staff were observed to be taking on more clinical duties such as giving injections to patients.

**Table 2:** Facility characteristics

Classification	Facility A	Facility B	Facility C	Facility D
	Health centre	Health centre	Dispensary	Dispensary
Monthly workload on selected indicators^a^				
Outpatient attendances	1953	882	1169	571
Outpatient confirmed malaria cases	39	314	475	18
Antenatal care attendance	328	67	91	70
Laboratory tests per month	1333	674	669	–
Staffing				
Clinical (clinical officers, nurses, lab techs)	9	5	6	3
Non-clinical staff (counsellors, peer educators)	3	5	5	2
Support staff (data clerks, and drug dispensers)	3	2	3	3

^a^Data obtained from the DHIS2 and represent average monthly workload in 2015. https://hiskenya.org/dhis-web-commons/security/login.action

### Recording malaria diagnosis data at frontline health facility

#### Malaria diagnosis data collected and reported at frontline health facilities

At the time of this study, malaria diagnosis data were supposed to be captured in four registers (Table 3): Outpatient (Under 5) morbidity register; Outpatient (Over 5) morbidity register; Laboratory register; and AL/RDT register. The AL/RDT register was designed to collect malaria programme specific data, and the rest to collect a range of health and service delivery data for various diseases, conditions and programmes. Ideally, each of these four registers should be completed at the time of service delivery; and each had instructions which health workers were supposed to adhere to when recording data. At the end of the month, malaria data recorded in the four registers were supposed to be collated and entered into six monthly reporting forms which are completed in duplicate; one to be submitted to the sub-county and the second retained at the health facility level for record purposes (Table 3). Facility managers were charged with the responsibility of ensuring that all monthly reports were completed, and that these were submitted to the respective sub-county health records offices by the 5th of every month for data entry into the DHIS2.

**Table 3:** Malaria diagnosis data recorded and reported at frontline health facilities

Register	Malaria data collected	Monthly summary form	Malaria data reported
1. Outpatient (Under 5) register	Malaria cases diagnosed < 5	1. Outpatient morbidity report < 5	Suspected malaria
	Malaria cases treated in < 5		Confirmed malaria
2. Outpatient (Over 5) register	Malaria cases diagnosed > 5	2. Outpatient morbidity report > 5	Suspected malaria
	Malaria cases treated > 5		Confirmed malaria
			Malaria in pregnancy
3. Laboratory register	Confirmed malaria cases	3. Laboratory workload summary report	Malaria Bs: < 5 (total examined and total positive)
	Negative malaria cases		Malaria Bs: >5 (total examined and total positive)
			Malaria RDTs: total examined and number positive
4. AL/RDT register	Microscopy (positive cases)	4. Monthly summary report for malaria medicines	RDTs: number positive and number negative
	Microscopy (negative cases)		Microscopy: number positive and number negative
	RDT (positive cases)		Total examined: microscopy and RDTs
	RDT (negative cases)		
		5. Annual work plan	Fever cases tested positive
		6. Facility data consumption request form	RDTs: patients < 5 (tested and confirmed)
			RDTs: Patients 5–14 years (tested and confirmed)
			RDTs: patients > 14 years (tested and confirmed)
			Microscopy: patients < 5 (tested and confirmed)
			Microscopy: Patients 5–14 years (tested and confirmed)
			Microscopy: patients > 14 years (tested and confirmed)

#### Recording malaria diagnosis and treatment data at frontline health facility

**Fig. 2:**
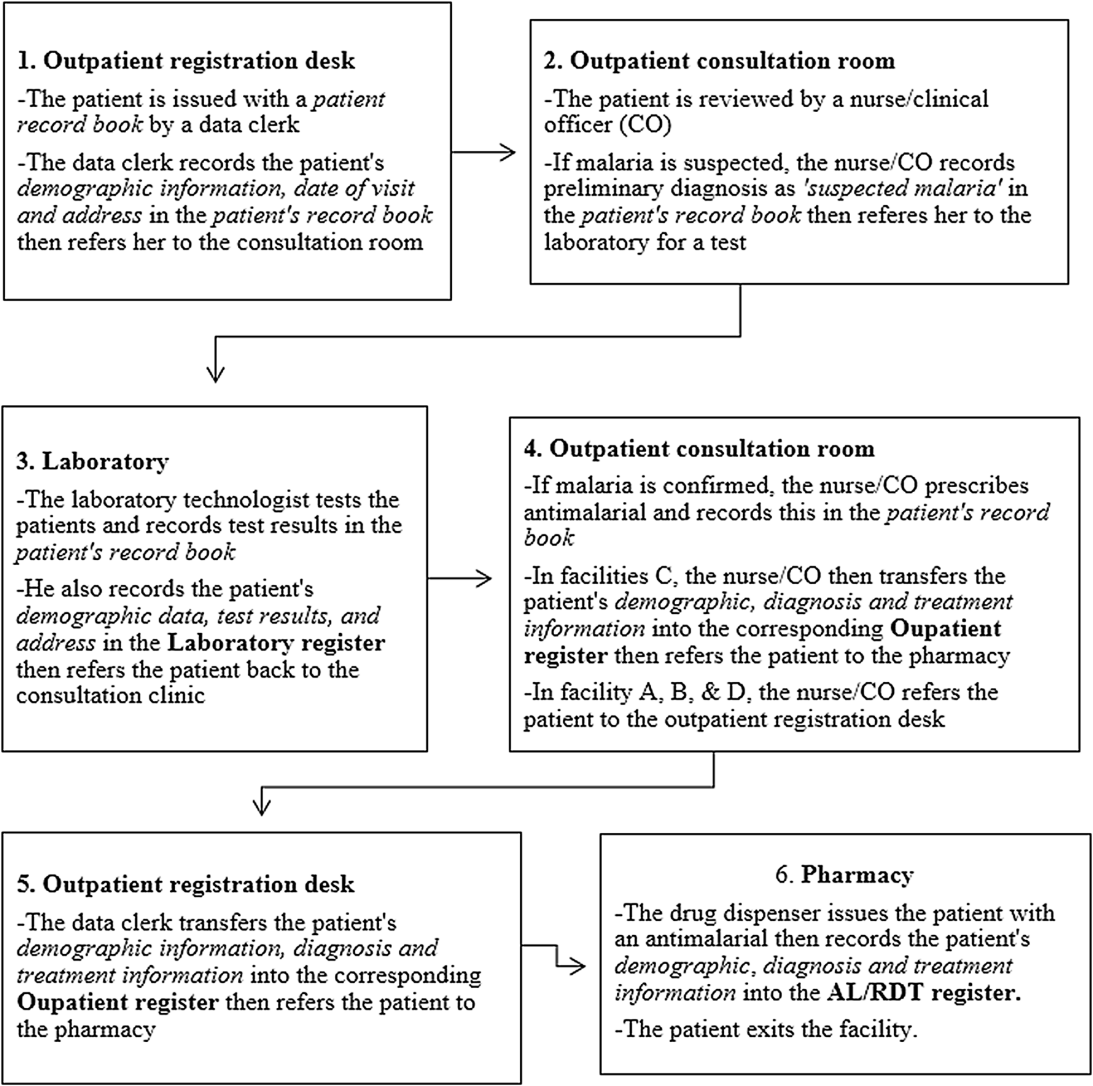
Malaria outpatient flow process and data recording

Figure 2 outlines the steps involved in recording malaria diagnosis data in the four studies facilities. Ideally, any suspected malaria case visiting the health facility should be reported to the outpatient registration desk where they are registered and issued with patient record books. From the registration desk, the patient is referred to the outpatient consultation clinic where he/she is reviewed by a nurse/clinical officer. If malaria is suspected, the patient is referred to the laboratory for a malaria test. From the laboratory, the patient returns to the outpatient consultation room where the nurse/clinical officer prescribes the recommended treatment then refers the patient to the pharmacy to collect their prescribed treatment. Each step in this process should be accompanied by a data record (Fig. 2).

#### Variations in malaria diagnosis data recorded in registers

**Fig. 3:**
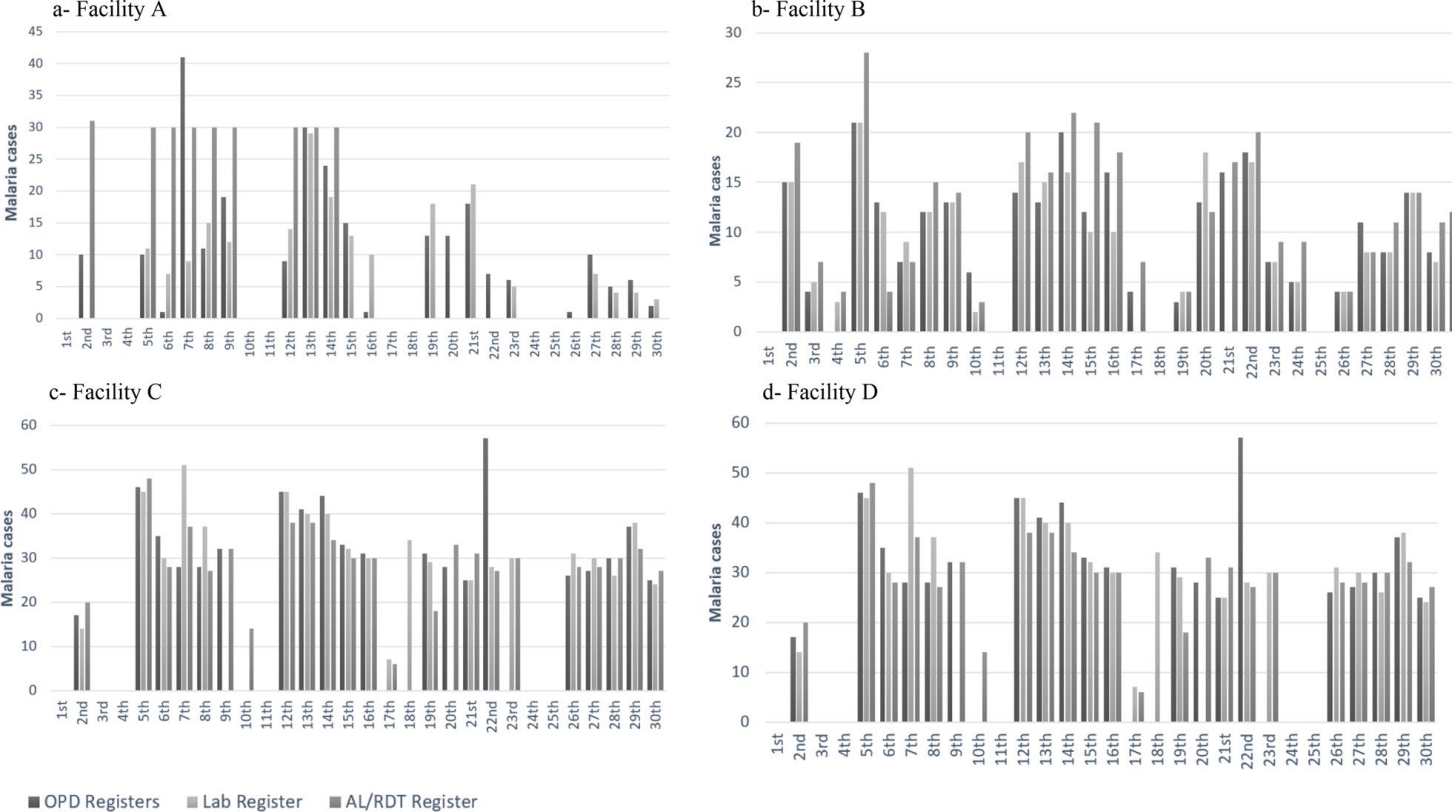
Malaria data recorded in primary registers in January 2015

If the standard outpatient visit process described in Fig. 2 were followed, then every confirmed malaria case should be captured in one of the two Outpatient registers, as well as in the Laboratory register, and the AL/RDT register. To explore whether or not this was the case, daily malaria diagnosis data recorded across the three service delivery areas (outpatient clinic, laboratory and pharmacy) in each of the four facilities were examined. The total number of malaria cases recorded on each day of the month in the Laboratory and in the Outpatient registers were compared with the total number of malaria cases which were recorded as having been issued with AL each day in the AL/RDT register in the month of January 2015 (Fig. 3).

Of all four facilities, only Facility D had relatively consistent data across the registers, and even in this facility on 5 of the 16 days (almost a third) for which there were data, there were discrepancies (see Fig. 3). There were considerable discrepancies in malaria cases recorded in the registers in the other three facilities on almost all days of the month (see Box 1).

These daily variations and inconsistencies in reporting within and among the registers in each facility were concealed in the monthly reports from the health facilities to the sub-county. For example, despite variations in facility B data (including missing laboratory data on 17th and 21st), their monthly reports indicated that the total number of confirmed malaria cases recorded in the outpatient registers were equivalent to confirmed cases in the laboratory (Table 4). This may be misinterpreted to mean that each confirmed malaria case recorded in the Laboratory register was also recorded in Outpatient registers which was not the case.

**Table 4:** Confirmed malaria cases reported in January 2015

Reporting form: Jan 2015	Facility A	Facility B	Facility C	Facility D
Outpatient morbidity reports	214	295	675	45
Laboratory report	205	295	660	Missing

**Box 1:** Examples of discrepancies in malaria data recorded in registers

Figure 3a	Figure 3b
– Cases treated for malaria consistently higher than outpatient and lab confirmed cases between 2nd and 14th – No cases recorded as treated in AL/RDT register between 15th and 30th despite lab and OPD recording cases – Outpatient confirmed cases higher than AL/RDT and Lab cases on 7th. Lab cases also fewer than AL/RDT cases	– Cases treated higher than outpatient and laboratory confirmed cases on 2nd, 5th, 15th, and 24th – Laboratory data missing on 17th and 21st – Outpatient data missing on 4th – Outpatient and laboratory confirmed cases higher than AL/RDT cases treated on 6th – OPD cases higher than cases recorded in the laboratory and AL/RDT register on the 27th
Figure 3c	Figure 3d
– Laboratory data missing on the 9th and 20th – Outpatient confirmed cases double number of laboratory and AL/RDT treated cases treated on the 22nd – Outpatient confirmed cases and AL/RDT cases treated missing on the 18th – Outpatient cases missing on the 17th and 23rd – Laboratory and outpatient confirmed cases missing on the 10th	– No cases recorded in the outpatient registers on the 5th, 7th, and 9th – Cases recorded in outpatient and AL/RDT registers higher than outpatient cases on 8th and 21st – Cases recorded in outpatient and AL/RDT registers higher than those recorded in lab register on 28th

Similarly, despite some of the variations pointing to the possibility of malaria being treated on clinical suspicion without a diagnostic test, (such as instances where the number of cases captured in the AL/RDT register were higher than those captured in the other registers), none of the four facilities reported any clinical malaria case in their outpatient morbidity reports. Health workers in all four facilities and those attending preliminary feedback meetings acknowledged that discrepancies indeed existed between malaria data recorded in primary registers and aggregated monthly reports.

*“These variations are there. You are just right. We have even tried to compare MOH 705A plus MOH 705B [outpatient morbidity reports] and MOH 706 [laboratory report] … We found out that the data was not the same in most facilities …”* Sub-county manager, Feedback meeting

Observations and interviews in the four study facilities revealed that these data quality problems were rarely caused by health workers deliberately manipulating their data. Rather, they were influenced by the broader context in which data collection and service delivery in general, took place. These issues are explored next.

### Practices and processes that shape data recording at frontline health facilities

Three key factors, spanning a range of system hardware issues emerged from the data as being central to the practices and processes of malaria data generation at frontline health facilities. These relate to: human resource shortages (and use of untrained support staff); the organization of service delivery; the nature of the data collection and reporting tools as well as the production and distribution of these tools (data collection tool stock-outs). Health workers used their systems software to address these systems hardware deficiencies, and in the process kept the system functional but with various outcomes for the quality of routine data produced.

#### Human resource shortages: informal task shifting and the role of untrained support staff

All four health facilities were understaffed. Due to the absence of trained health records and information officers, data collection responsibilities in the outpatient and pharmacy departments in all of the facilities were primarily delegated to support staff, none of whom had received any formal training in data recording.

*“We don’t have a registry clerk and I am only doing to help. It is not my profession. If someone came and asked me questions [about data], I wouldn’t be in a position to respond to him. I have never studied anything to do with data or registry. I am just here to assist.”* Support staff, IDI

Due to the lack of formal training, these support staff relied mainly on their experience acquired over time to fulfil their data collection roles. Some of their practices strengthened the data collection process. For example, support staff working in facility D devised a recording strategy where no drugs were issued to patients without an outpatient visit number being present in the patient record book (serving as proof that their data had been transferred into the outpatient register). This recording strategy may have contributed to the consistencies seen in the data from this facility (see Fig. 3). However, some practices undermined the process of accurate data recording. For instance, observations in outpatient departments in facilities B and D revealed that whenever diagnostic information in a patient’s record book was illegible, rather than seek clarifications from the prescribing health worker, support staff used their experience to determine the ‘correct’ diagnosis and recorded this interpretation in the Outpatient registers. It is unclear whether their interpretations were correct, but the data recorded in the Outpatient registers and subsequently reported at the end of the month hide these interpretations and any differences with nurses/clinical officers’ records. Data quality problems in facility A (Box 1) were also attributed to the inconsistent use of the AL/RDT register by the member of support staff working in the pharmacy during this period.

*“As a matter of fact, that register wasn’t being used at the beginning of last year. Sometimes the drugs were being issued but the register was not being used consistently. That is why you see we have dispensed AL on a daily basis but when you check the register, it is not recorded”* Health worker, feedback meeting

Some health workers and sub-county managers acknowledged that the involvement of support staff in the data collection process possibly undermined data quality, an issue that has been documented in data quality audit reports in Kenya [13].

*“We have been using support staff to fill these reports. At the end of the day, whatever these support staff will fill is what you will get. So garbage in garbage out. At the end of the day, we will complain that our data is not of good quality”* Health worker, feedback meeting

Despite the critical role they played in the data collection process, and recognition of their limited capacities in data recording, this cadre of staff rarely got an opportunity to attend any training on data collection. These staff were poorly remunerated, overworked and paid irregularly. For example, at the start of field work, support staff and locally recruited laboratory technologists working in facilities B, C and D had not received their salaries for over 3 months. To cushion them from salary delays, support staff in these facilities adopted small income generation strategies such as: stocking and selling drugs which were unavailable in the facility’s pharmacy to patients at a fee (facility D); procuring their own reagents and conducting laboratory tests at a fee (facility B, C and D); and charging patients for certain services (facility C, and D). Some members of the support staff sought additional employment to cope with delays in their regular employment payments. For example, one of the data clerks worked on locum in a nearby health facility without the approval of the facility manager. This particular member of support staff was responsible for data collection in outpatient clinics. His absence therefore increased workload for the remaining support staff in this facility.

#### Organization of service delivery

Patients reporting to each of the four health facilities with suspected malaria were supposed to go through the outpatient visit process described in Fig. 2. In practice, this standard malaria outpatient visit process was not always followed. To manage workload, outpatient consultations were also provided in a variety of other locations in each facility: the HIV/AIDS consultation clinic (facility B); the outpatient waiting bay (facility C); the examination room (facility D); and in the ANC clinics (facility A, B and C). While the details of patients seen in service delivery areas other than the outpatient consultation room in facility A, B and D were always recorded in Outpatient registers, this was not always the case in facility C where Outpatient registers were located inside the outpatient consultation room (Fig. 2). As such, details of patients who were attended to in the outpatient waiting bays were not always recorded in the outpatient register. However, these patients’ details were always recorded in the laboratory register (if tested in the laboratory) and in the AL/RDT register (if issued with AL). The facility manager explained that this practice possibly contributed to some of the data quality problems (such as missing data in Outpatient registers) in this facility.

*“…in the late afternoon, you will see people being sent to the laboratory for tests from the waiting bay. The patient will go to the laboratory and will be prescribed a treatment. The patient will go straight to the pharmacy without his details being recorded in the [Outpatient] register.*” Health worker, IDI

Similarly, apart from the laboratory, malaria RDT tests were also conducted in other locations: the Voluntary Counselling and Testing (VCT) clinics (facility B, C, and D); outpatient consultation rooms (facility B and D); and the HIV/AIDS clinic (facility B). In facilities C and D, the results of malaria tests conducted outside the laboratory were usually recorded in the Laboratory register. However, in facility B, the results of tests conducted outside the laboratory were recorded in several improvised registers which were inconsistently used, contributing to data quality problems:

*“…we have put a book [improvised register] there though some people will assume it’s not there and just do the tests only. It mostly happens to clients being seen at night when somebody uses RDT and once he has given the drugs that’s all”* Health worker, IDI

In addition, health workers in facility B explained that there were cases when patients were referred to the laboratory from private pharmacies for malaria tests. Data from these patients were captured in the laboratory registers but not outpatient and AL/RDT registers since such patients exited the facility without going through the pharmacy or outpatient clinics. Further potential explanations for missing data in registers were patients with confirmed malaria cases leaving the facility without their details being entered in the outpatient or AL/RDT registers; a practice that GO and SZ observed in the field.

#### Influence of data collection tools

The design of registers coupled with unclear or missing instructions for data recording created confusion and undermined the standardization of data collection practices in all four facilities. For example, instructions available in the Outpatient registers for recording data in the diagnosis column stated that: ‘*the provisional or final diagnosis from the clinician must be recorded in this column’.* This meant that both clinical (suspected) and confirmed cases of malaria were recorded in the same column alongside other diagnoses. In response, health workers in all four facilities adopted local recording strategies which enabled them to navigate through these challenges. The coping strategies varied within and between the four facilities. To distinguish between *clinical* and *confirmed malaria cases,* staff recording data in facility C used the comments section of Outpatient registers to record ‘*no test’* (if malaria was treated clinically); *‘RDT pos/Bs*++*’* (for confirmed malaria cases) or ‘*RDT neg’* (for negative malaria cases). In facility B, they recorded clinical malaria cases as ‘*cl. Malaria*’ in the diagnosis column. In facility A, a red pen was used to record confirmed malaria cases in Outpatient registers. In facility D, all malaria cases were simply recorded in the diagnosis column as ‘malaria’. The facility manager explained that in this facility, they rarely treated malaria clinically. Although Outpatient morbidity tally sheets designed to be completed alongside Outpatient registers allowed health workers to separately record *clinical* and c*onfirmed malaria* cases, in practice, these sheets were only used in facility A. Health workers in the other three facilities perceived that these tally sheets increased their workload, were difficult to implement due to the multiplicity of individuals involved in provision of outpatient consultation services, and that their use contributed to confusion and data quality problems.

*“We stopped using tally sheets because it [data recorded] was never the same with the [outpatient] register. When someone is in the mood, he will tally. When he is not in the mood, he doesn’t tally. So by the end of the day, that data will not tally. So we opted to use the register. So from that register is where we tally [extract data]”.* Health worker, IDI

For the Laboratory register, while standard guidelines required laboratory technologists to record malaria parasite density and type of malaria parasites (reported as xxx number of parasites per 200 white blood cells) [20], there were no separate columns for recording this information in the register. Only the laboratory technologists in facility A and B recorded malaria parasite density count and type of parasites seen. They used the results column to record these data but argued that collecting this information unnecessarily increased their workload since it did not improve malaria management as one of them observed during an interview.

*“…as much as this system of reporting gives you the parasite load per millilitre (ml) of blood, there is no specific guideline saying that this number of parasites in a ml of blood we can now term as severe malaria”* Health worker, IDI

The Laboratory register was designed to capture malaria diagnosis information (suspected and confirmed malaria cases), however, similar information was captured in the AL/RDT register resulting in unnecessary duplication and data burdens, a key concern for health workers in all four facilities as described in a previous paper [21].

Throughout the study, health workers complained about the poor design of data collection and reporting tools designed by national level managers who were described as oblivious to service delivery or data collection realities on the ground.

*“I think the people who prepare these registers are not experienced in terms of sitting in a clinical area and seeing what is needed and what is not needed. This is someone who is very learned. They are put in a hotel and then they do these things. I wish they could get our views… We make some recommendations and then it goes up like that. So, they know that this can be done, and this cannot be done.”* Health worker, IDI

#### Stock-out of registers and reporting tools

Shortage of standard data collection tools also had an influence on data collection practices in all four facilities. There was a nationwide shortage of data collection tools during this study. A review of facility records at the beginning of fieldwork in January 2015 showed that some of the tools had been out of stock for over a year. Stock-out of data collection tools was linked to the lack of clarity on the roles of county and national government in tool development and printing post-devolution of health service management function.

*“The national [government] is supposed to supply the counties with the tools but now because of devolution you know there is that push and pull. The national [government] now say that it’s counties mandate to provide the tools. The county also says that the national have not provided us with funds to bring these tools.”* Sub-county Manager, IDI

In the absence of standard data collection tools, health workers used various non-standard registers to record service delivery data. For example, Inpatient registers were used in place of Outpatient and Laboratory registers in facility B. In facility A, a simplified version of the Laboratory register developed by laboratory technologists was used to record laboratory data. Inpatient registers were also used to record laboratory and outpatient data in facility C. In all instances when non-standard data collection registers were used to record data, health workers only included in these improvised registers the data columns that were useful for the compilation of the monthly reports required by the sub-county. For example, the improvised AL/RDT register (an exercise book) in use in facility C only captured data on the number of AL doses dispensed, the only information required for reporting at the end of the month. Other data categories such as patient’s weight, which were important in determining the correct dose of AL but were not transferred to any of the reports at the end of the month, were not included in the improvised register. Similarly, the improvised Laboratory register in use in facility A only had 10 out the 25 columns contained in the standard register. These 10 were the ones which were required when compiling monthly reports. This suggests that improvisations were mainly motivated by the need to fulfil reporting obligations. Irrespective of whether the standard tools were available or not, submission of monthly reports to the sub-county was compulsory. Health workers were aware of this requirement hence the common practice of developing and using improvised tools when standard registers were unavailable.

*“When it comes to end month, you are expected to submit a report. You know reports can only be generated from these documented data. So, when somebody comes and asks did you submit your report? Then you say yes. Where is the source of the report? Then you give this one”* Health worker, IDI

## Discussion

The data presented in this study have demonstrated that routine malaria data generation at health facility levels took place in a difficult environment that was characterized by various systems hardware constraints such as shortages of human resources, stock-out of data collection tools, and poorly designed tools. These challenges are typical of primary health care service delivery in many countries in sub-Saharan Africa [22–24]. The challenges had a direct influence not only on malaria data generation, but also on service delivery practices in general. Health workers had little or no power to influence many of the systems hardware challenges that they faced (e.g. shortages of trained staff, lack of appropriate tools and shortage of data collection tools); but they drew on their interests and values (systems software) (Fig. 1) [15] and exerted their ‘power with’ and ‘power to’ (Table 2) [16] to develop a range of local coping strategies that had a range of consequences for the outcome of the data collection process. These local coping strategies were motivated by the shared need to keep the system ‘functional’ but had unintended consequences in some instances.

Shortages of adequately trained health professionals and technical support staff is a well-recognized problem in many low-income settings [25]. Across all of the health facilities, facility managers and health facility management committees worked together (exerting their ‘power with’) to address staff shortages by spending their discretionary funds on employing support staff. However, these support staff were untrained, overworked, and rarely accorded an opportunity to attend sub-county level training. Although delegating data collection roles to them freed up time for health workers to concentrate on other service delivery areas, at times, what they recorded in registers did not accurately represent what nurses/clinical officers had written/not written in patients’ record books. Furthermore, support staff were poorly paid and often experienced salary delays which affected their morale. They continued to perform their data collection responsibilities but demonstrated their dissatisfaction through exercising their power to act in strategies such as delayed completion of reports and charging for services which should have been free; actions which had detrimental effects on malaria data generation.

A number of studies conducted in sub-Sahara Africa have documented mixed outcomes from delegating certain tasks to untrained staff [26]. For instance, Mpofu et al. found that shifting monitoring and evaluation duties from nurses to other professionals improved data quality, management and reporting, and also freed up time for nurses to concentrate on other duties in Botswana [27]. By contrast, in Malawi, managers raised concerns that lay health workers were posing as doctors and providing services that were beyond their scope [28]. Although task shifting has been promoted as a possible strategy for addressing staffing challenges in the region, and improving service delivery [29], the data from this and other studies suggest that such strategies would require the provision of training opportunities, a good working environment, adequate support supervision and effective regulatory frameworks, to ensure both effective service delivery and adequate data recording and reporting practices [30].

The results of this study have also shown that there was a severe stock-out of standard data collection tools in all four facilities at the time of this study which had a bearing on recording practices. Stock-out of data collection tools is a recurrent problem in Kenya and other settings across sub-Sahara Africa [5, 6, 31] and point to weak supply chain management at national level. When no standard registers were available, health workers used their power to act and developed their own improvised registers which sustained the data collection process but had varied consequences on the outcome of the process. The use of improvised tools allowed health workers to continue fulfilling various accountability requirements, but undermined standardization of data collection as has been noted elsewhere [31].

A specific element of hardware found to have a direct influence on data generation at frontline health facilities was the design of data collection registers and instructions for data collection. Lippeveld et al. observed that “*the quality and ultimate use of the data collected through routine information systems will depend substantially on the relevance, simplicity and layout of the data collection instruments”* [32]. This study found that poor design of data collection tools led to variability in recording and reporting practices which undermined standardization and possibly contributed to poor data quality. Such issues have been reported in previous studies [6, 12, 31]. In addition, poor layout of Outpatient registers made it difficult for health workers to segregate clinical and confirmed malaria cases. This problem possibly contributed to the misreporting of malaria cases that has been found in recent assessments of routine malaria data [11, 12]. Although data quality audits recommended training for health workers to eliminate these confusions [13], this study found that health workers’ inability to separate clinical from confirmed malaria cases are more likely to be caused by the poor design of the Outpatient registers. These findings also point to a limitation of current data quality audit tools which are very focused on assessing the quantitative aspects of data quality, potentially failing to reveal the true causes of poor data quality. This possibility was also noted in a recent review of the data quality assessment methods employed in public health information systems [14].

The recording and reporting tools that were in use at the frontline health facilities during this study were developed at the national level by managers who were perceived to be oblivious to the service delivery or data collection and reporting realities on the ground. These managers used their power over the process to decide on indicators, data collection tools, and data collection procedures which health workers at the frontline were required to adhere to when collecting and recording data. However, how these tools were used or rules followed was dependent on health worker’s ‘power to’ or their discretionary power, which refers to the ‘*power exercised by those at the frontline of service delivery t whose actions cannot be fully controlled by central actors’* [30]. For example, health workers used their power to act to determine which of these tools to use (e.g. decision not to use tally sheets in facility B, C and D); and what to record (e.g. only 10 columns included in the improvised laboratory register in facility A). In the Kenyan context, managers at higher reporting levels only received aggregated monthly reports and so these local variations in recording and reporting practices remained concealed in facility records. As observed by Chaulagai et al. managers and other consumers of routine data became ‘passive consumers of information’ whose quality or even source was unknown to them [33]. Some authors have argued that involving frontline staff in the development of data collection tools can significantly improve the relevance and utility of these tools to data producers [32–36]. The findings from this study would support this approach.

### Limitations

This study was conducted in a limited geographical area and in a small number of health facilities. As such, the results of this study may not be generalizable to other areas of Kenya. However, to improve analytical generalizability, the paper drew on theory to explain practices and processes that shape routine health data generation at frontline health facilities. To improve validity, this study relied on multiple approaches to data collection which enabled triangulation between data sources. In addition, feedback meetings also enhanced the validity of this study as participants had an opportunity to listen and provide feedback on preliminary findings. The use of quantitative data obtained from records reviews also strengthened descriptive and interpretive validity of the study.

## Conclusion

This study has shown that most of the challenges encountered by health workers in routine malaria data generation at the health facility level have their roots in wider system issues and at the national level where the framing of indicators and development of data collection tools takes place. These challenges cannot therefore be addressed by HIS or disease specific interventions per se as studies of routine health information systems in sub-Saharan Africa have always recommended. Fiddling with one component of the system, e.g. changing the design of data collection tools (which was a problem in this study), while ignoring broader systemic issues such as human resource shortages are unlikely to result in sustainable improvements in the outcomes of the data collection process. More importantly, this study has demonstrated the importance of systems ‘software’ (relationships and contestations, motivations and interests etc.) in shaping how those at the frontline of malaria data generation responded to various health system constraints, demonstrating resilience in keeping the system ‘functional’ but with unintended consequences for data quality.

## Data Availability

The datasets generated and analysed during the current study are not publicly available due institutional rules and regulations but are available from the corresponding author on reasonable request.

## Abbreviations

HIS: health information system
DHIS2: District Health Information Software
DQAs: data quality audits
AL: artemether–lumefantrine
RDT: rapid diagnostic test
IDI: in-depth interview
VCT: voluntary counseling and testing
